# The YMCA/Steps Community Collaboratives, 2004-2008

**Published:** 2009-06-15

**Authors:** Ellen S. Jones, Katie Adamson, Dennis Shepard, Alyssa Easton

**Affiliations:** National Association of Chronic Disease Directors; YMCA of the USA, Chicago, Illinois; YMCA of the USA, Chicago, Illinois; Centers for Disease Control and Prevention, Atlanta, Georgia

## Abstract

Since the YMCA/Steps National Partnership began in 2004, the collaborative approach has built local synergy, linked content experts, and engaged national partners to concentrate on some of the most pressing health issues in the United States. Together, national and local partners used evidence-based public health programs to address risk factors such as poor nutrition, physical inactivity, and tobacco use. This article describes the YMCA/Steps National Partnership and focuses on the experiences and achievements of the YMCA/Steps Community Collaboratives, conducted with technical assistance from the National Association of Chronic Disease Directors between 2004 and 2008. We introduce some of the fundamental concepts underlying the partnership's success and share evaluation results.

## Background

In 2004, a unique partnership was formed between the YMCA of the USA and Steps to a HealthierUS. This partnership advanced community health promotion and became a model for improving health at the grassroots level.

YMCAs are at the heart of community life in the United States: 70 million households are within 3 miles of 1 of the 2,686 YMCAs. The organization serves more than 21 million people, of whom 10 million are younger than 18 years, and offers activities such as camping, youth sports, and after-school programs.

Steps to a HealthierUS (Steps Program), a community health initiative that is part of the US Department of Health and Human Services and is coordinated by the Centers for Disease Control and Prevention (CDC), focuses on preventing the leading causes of death and disability — heart disease, cancer, obesity, diabetes, and asthma. The Steps Program (now called the Healthier Communities Program) not only addresses these conditions directly but encourages communities to improve policies and environmental factors to enhance supports for healthy choices so that communities improve health outcomes and reduce the economic costs of poor health due to chronic conditions (1,2).

A centerpiece of the Steps Program was a cooperative agreement program through which states, cities, and tribal entities receive 5-year grants of $500,000 to $1.5 million to prevent chronic disease. In 2003, the Steps Program allocated $13.6 million to grantees representing 24 communities. In 2004, the Steps Program granted $35.8 million to increase funding to the existing communities and to fund an additional 16 communities. These 40 funded Steps communities began to develop and implement community action plans to reduce health disparities and promote quality health care and prevention services.

As of 2004, 2,594 YMCAs were already working within communities throughout the United States. With the shared commitment expressed in the YMCA mission statement to "build healthy spirit, mind, and body for all," YMCAs lend the partnership their expertise in cultivating community relationships that ensure programs, services, and systems are integrated into all aspects of life and reach those most in need. Through Activate America, the YMCA's approach to addressing the nation's growing health crisis, Steps communities gained the YMCAs' access to community leaders, enabling them to assess their communities' health needs and work toward improved health for their residents (3,4).

In 2004, the Steps Program selected YMCA of the USA as its national partner to increase the impact and reach of the Steps cooperative agreement. The Steps Program awarded the YMCA of the USA $2 million over 4 years to build lasting partnerships between local YMCAs and the 40 Steps communities. Each year between 2004 and 2008, YMCAs could apply for mini-grants to work with a Steps community to form a local partnership known as a YMCA/Steps Community Collaborative. The mini-grants facilitated the development of community action plans that focused on local needs and drew on local resources.

When given the right tools and support, families, businesses, schools, worksites, and faith-based organizations can work together to set the stage for healthier choices. At the same time, national leadership can create a support network, leveraging local efforts so communities can learn from each other's experiences (5).

Acting separately, YMCAs and Steps communities have been successful, but acting in partnership, results have been magnified with better planning, outreach, knowledge and skills, leadership abilities, and sustainability (6). We introduce some of the fundamental concepts underlying the partnership's success, highlight the partnership's accomplishments, and share evaluation results.

## Implementation

During 4 years, the projects confirmed that lasting changes in support of public health often occur at the community level with the engagement of local leaders and stakeholders (7). The collaborative projects focused on grocery stores, school nutrition and physical education, mass transit, walking and biking trails, health care providers, and collaboration with local government to address health-related policies and programs offered through venues such as parks and recreation departments.

Formal agreements between the national partners and lessons learned from the experiences of the collaboratives were systematically collected and shared with the 40 YMCA/Steps Community Collaboratives through local programs, services, and outreach. The national scope of this project strengthened the momentum of local collaboratives as they gathered baseline data to determine where their efforts had the best chance of success.

## Steps to Success

Although the projects involved 40 communities with multifaceted components, some common steps to success included the following:

- Engaging a multidisciplinary, culturally diverse group of local stakeholders.
- Developing an action plan driven by the community.
- Ensuring that evaluation strategies were included from the beginning.
- Recruiting local partners and identifying local resources to ensure sustainability.
- Enlisting the support of national partners such as the YMCA/Steps National Steering Committee and CDC for substantial technical assistance with implementation.
- Implementing the action plan and ensuring that it was driven by the community players that were vital in its development.
- Revisiting the plan and making adjustments and revisions based on evaluation results and community feedback.

The potential pitfalls to these steps to success included the following:

- Failure to engage a diverse group of stakeholders, resulting in a few people "telling" the community what it needs.
- Insufficient attention to sustainability of local programs in terms of financial and personnel support.
- Evaluation measures that are not relevant or timely to the community action plan.

## Effect

Over 4 years, the 40 community collaboratives developed community action plans that connected YMCA work with local public health and community efforts (Table).

Changes in the health of future generations will depend largely on our ability to reduce risk factors for chronic diseases through better nutrition, physical activity, and prevention of diabetes, heart disease, and cancer. The partnership between YMCA of the USA, Steps to a HealthierUS, and the National Association of Chronic Disease Directors has allowed these organizations, local YMCAs, and Steps communities to learn lessons, gain relevant experience in improving community health, and build a base for collaboration through which families and individuals learn about being healthy.

In the fourth and final year, a qualitative assessment was conducted. The following discussion highlights some of the results from the survey used to assess this partnership. Complete results of the survey can be found at www.ymca.net/downloads/aa_steps_evaluation_report.pdf.

**Figure 1.**
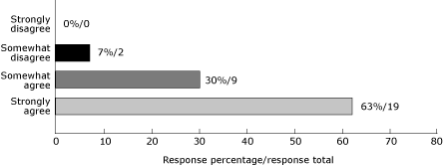
Assessment of the YMCA/Steps National Partnership's success in strengthening public health partnerships. The National Partnership's steering committee members responded to the statement, "The YMCA/Steps initiative encouraged us to develop new partners or nurture relationships."

The national YMCA/Steps partnership was guided by a steering committee that provided technical assistance, links to materials, and expertise. Steering committee members were engaged throughout the project, and their views are summarized below and in Figure 1:

- Participation on the steering committee was beneficial to each agency (92% agreed).
- Technical assistance was valuable to participants in the project (85% agreed).
- The project encouraged steering committee members to develop new partnerships and nurture existing ones (92% agreed).

**Figure 2.**
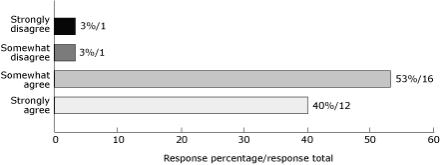
Assessment of the YMCA/Steps National Partnership's success in improving familiarity with chronic disease prevention. The National Partnership's steering committee members responded to the statement, "I am now more familiar with effective practices in chronic disease prevention."

At the project's conclusion, representatives from the 40 community collaboratives were surveyed. The results of the national questionnaire, which had an 80% response rate, indicate a strong sense of accomplishment and sustainable policy and environmental changes (Figure 2). Respondents observed the following:

- They were more familiar with effective practices in chronic disease prevention than at the project's inception (93% agreed).
- They had been able to implement evidence-based programs (87% agreed).
- Outreach to underserved populations had increased (93% agreed).
- Policies and environments had changed in their communities as a result of the project (77% agreed).

Many respondents said the project had resulted in positive environmental or policy changes at external sites: worksites (48% perceived positive changes), schools (45%), and other state or recreation agencies (41%). Some of the communities have also received complementary funding for the Pioneering Healthier Communities (55%) or REACH 2010 (9%) programs. Most of the YMCAs (73%) were participating in YMCA Activate America activities to implement internal systems strategies to improve health.

Some of the accomplishments the partners said they were proudest of included

- Providing more nutritious food at YMCAs, day care centers, schools, and recreation centers.
- Incorporating health promotion in after-school programs.
- Gathering local businesses engaged in community health and supporting community events.
- Connecting health care systems to YMCA and community efforts.
- Building a national network of organizations dedicated to promoting the YMCA/Steps National Partnership model of collaboration.

## Conclusion

As the YMCA/Steps Community Collaboratives model is adapted for use in other communities, the focus should remain on building capacity and sustainability. These collaborative projects can create a ripple effect by garnering resources such as expertise from public health officials and national organizations and seed funding. Matching resources help sustain and grow efforts locally. One measure that demonstrated local commitment to the partnership was the percentage of awarded funds used to enhance YMCA/Steps Community Collaboratives over the 4 years — 131% of funds awarded. Although this is at best a proxy measure to demonstrate local commitment, it provides tangible evidence that the partnership was willing to provide additional local assets to build a healthier community. These changes are small investments that can build community-based health improvements in systems, policies, and environments that multiply over time so that they will have a lasting effect. Systems changes have taken hold that will promote the YMCA's mission and support public health efforts to establish wellness and health opportunities at the community level.

## Figures and Tables

**Table.** Examples of Policy and Environmental Approaches, YMCA/Steps Community Collaboratives, 2004-2008

**Change Intervention**	**Worksite**	**School**	**Health Care**	**YMCA Healthy Families Programming**
**Policy**	Encouragement of exercise at work Coverage of health screenings by benefits plan	Changes in school vending Increase in school physical activity time	Acceptance of referrals from YMCA Prescriptions for physical activity	Improvement of vending and after-school nutrition policies Increased flexibility of facility hours to meet community needs
**Healthy environment**	Organization of corporate exercise Engagement of business leaders	Enhancement or establishment of community or school walking trails Engagement of YMCA and public health staff in schools	Distribution of healthy cooking information at doctors’ offices Inclusion of obesity curriculum in health care settings	Addition of pool lifts to increase accessibility for community residents with disabilities Alteration of trail environments to engage all ages
